# Efficacy and safety of duloxetine in painful diabetic peripheral neuropathy: a systematic review and meta-analysis of randomized controlled trials

**DOI:** 10.1186/s13643-023-02185-6

**Published:** 2023-03-21

**Authors:** Chung-Sheng Wu, Yu-Jui Huang, Yuan-Chun Ko, Che-Hsiung Lee

**Affiliations:** 1 Department of Primary Care Medicine, Shuang-Ho Hospital, Taipei Medical University, New Taipei City, Taiwan; 2 Department of Psychiatry and Psychiatric Research Center, Taipei Medical University Hospital, Taipei, Taiwan; 3 Department of Primary Care Medicine, Wan-Fang Hospital, Taipei Medical University, Taipei City, Taiwan; 4 Department of Plastic and Reconstructive Surgery, Division of Trauma Plastic Surgery, Chang Gung Memorial Hospital, No. 5, Fuxing St, Gueishan District 333, Linkou, Taiwan; 5 Department of Plastic Reconstructive, Tucheng Hospital, Tucheng Dist, New Taipei City, Taiwan; 6 Department of Biological Science and Technology, National Chiao Tung University, Hsinchu City, Taiwan

**Keywords:** Painful diabetic polyneuropathy, Duloxetine, Meta-analysis, Systematic review

## Abstract

**Background:**

Painful diabetic peripheral neuropathy (PDPN) is a key concern in clinical practice. In this systematic review and meta-analysis, we compared duloxetine and placebo treatments in terms of their efficacy and safety in patients with PDPN.

**Methods:**

Following the PRISMA guidelines, we searched the Cochrane Library, PubMed, and Embase databases for relevant English articles published before January 11, 2021. Treatment efficacy and safety were assessed in terms of pain improvement, patient-reported health-related performance, and patients’ quality of life.

**Results:**

We reviewed a total of 7 randomized controlled trials. Regarding pain improvement, duloxetine was more efficacious than placebo (mean difference [MD] − 0.89; 95% confidence interval [CI] − 1.09 to − 0.69; *P* < .00001). Furthermore, duloxetine significantly improved the patients’ quality of life, which was assessed using the Clinical Global Impression severity subscale (MD − 0.48; 95% CI − 0.61 to − 0.36; *P* < .00001), Patient Global Impression of Improvement scale (MD − 0.50; 95% CI − 0.64 to − 0.37; *P* < .00001), and European Quality of Life Instrument 5D version (MD 0.04; 95% CI 0.02 to 0.07; *P* = .0002). Severe adverse events were rare, whereas nausea, somnolence, dizziness, fatigue, constipation, and decreased appetite were common; approximately, 12.6% of all patients dropped out because of the common symptoms.

**Conclusions:**

Duloxetine is more efficacious than placebo treatments in patients with PDPN. The rarity of severe adverse events indicates that duloxetine is safe. When a 60-mg dose is insufficient, 120 mg of duloxetine may improve PDPN symptoms. Our findings may help devise optimal treatment strategies for PDPN.

**Systematic review registration:**

PROSPERO CRD42021225451

**Supplementary Information:**

The online version contains supplementary material available at 10.1186/s13643-023-02185-6.

## Background

Painful diabetic peripheral neuropathy (PDPN) is caused by chronic hyperglycemia and characterized by nerve damage and intolerable pain [1]. Approximately 50% of all patients with diabetes develop peripheral neuropathy [2], 10 to 26% of whom experience PDPN [3]. Clinical manifestations of PDPN include the neuropathy of distal lower extremities, which involves tingling, shooting pain, burning pain, allodynia, hyperesthesia, and other unusual sensations. Symptoms often deteriorate at night and affect sleep quality. Some patients may experience mood disorders, such as anxiety and depression [4–6].

The precise pathophysiology of PDPN remains debatable. A commonly accepted hypothesis is that distal nerve fiber damage would result in altered peripheral signaling and compensatory changes in the central nervous system, thereby disrupting the mechanisms underlying the inhibition of endogenous pain; this would promote the hyperexcitability and sensitization of pain-transmitting pathways and cause severe and persistent pain [4, 7].

The neurotransmitters serotonin (5-hydroxytryptamine) and norepinephrine help modulate nociceptive transmission by descending pain inhibitory pathways in the brain and spinal cord [8–10]. Duloxetine hydrochloride is a potent dual serotonin–norepinephrine reuptake inhibitor (SNRI); it modulates the mechanisms underlying PDPN [11]. SNRI antidepressants increase noradrenaline levels and target α2-adrenergic receptors in the dorsal horn of the spinal cord and the locus coeruleus. These pathways are highly efficacious against allodynia and hyperalgesia, which are associated with neuropathic pain. Serotonin may also enhance the inhibitory effects of noradrenaline in an auxiliary manner [12].

Currently, duloxetine and pregabalin are the only drugs approved by the US Food and Drug Administration (FDA) for treating PDPN. Pregabalin and duloxetine have been recommended as the first-line therapy in a total of 5 and 4 relevant guidelines, respectively. The 4 guidelines recommending duloxetine are the Neuropathic Pain Special Interest Group guideline (International Association for the Study of Pain), European Federation of Neurological Societies guideline, National Institute for Health and Care Excellence guideline (UK), and Canadian Pain Society guideline [13–17]. The additional guideline recommending pregabalin is the American Academy of Neurology guideline; the aforementioned difference between the 2 drugs in terms of recommendation stems from the fact that a limited number of duloxetine trials were graded as class I, and thus, data were regarded to be insufficient for level A recommendation for this drug [18, 19]. Nevertheless, duloxetine is not inferior to pregabalin in treating PDPN [20, 21]. To the best of our knowledge, no meta-analysis conducted after 2015 has evaluated the role of duloxetine in the treatment of PDPN [22]. Moreover, our knowledge regarding the clinical utility of duloxetine, different efficacies of its prescribed dose, and severity of associated adverse events in patients with PDPN remains limited. Therefore, in the present updated systematic review and meta-analysis of randomized-controlled trials (RCT; placebo-controlled), we evaluated the efficacy and safety of duloxetine in the treatment of PDPN. We extracted data from earlier studies in which relevant scales and questionnaires were used to investigate the optimal dose for treatment, the drug’s efficacy in improving pain and patients’ quality of life and the severity and occurrence of adverse events.

## Methods

### Study design and inclusion criteria

This systematic review and meta-analysis were conducted following the Preferred Reporting Items for Systematic Reviews and Meta-Analyses guidelines [23]. The study protocol was developed and registered in the PROSPERO database (CRD42021225451; January 9, 2021). The selection criteria were defined before the literature search and included the PICO components—problem/population, intervention, comparison, and outcome. We included studies on the comparison between duloxetine and placebo treatments in adult patients with PDPN due to diabetic polyneuropathy who presented with daily pain for > 6 months, in whom the symptoms started from distal extremities bilaterally, and whose weekly average visual analog scale pain scores were > 4. All doses and treatment durations of duloxetine were reviewed. The primary outcome measure was a reduction in patients’ weekly mean pain scores, assessed using an 11-point Likert-type scale, for 24-h average pain. The secondary outcomes included 24-h worst pain and night pain; numbers of patients with 30% and 50% reductions in 24-h pain scores [24]; and scores on the Brief Pain Inventory (BPI) scale (severity and interference) [25], Short Form-36 (SF-36) questionnaire [26], Patient Global Impression of Improvement (PGI-I) scale [27], Clinical Global Impression scale (CGI; severity subscale) [28], European Quality of Life Instrument 5D version (EQ-5D) [29], and Short-Form McGill Pain Questionnaire (SF-MPQ; sensory component) [30]. Adverse events and compliance were assessed in terms of the number of patients discontinued because of adverse events, number of patients with at least one adverse event or severe adverse event, and incidence rate of common adverse events. Non-RCTs, case reports, reviews, animal studies, letters to editors, and conference abstracts were excluded from our review. No restrictions related to trial duration were applied.

### Search strategy

We searched the Cochrane Library, PubMed, and EMBASE databases for relevant RCTs published in English before January 11, 2021. Keywords used for the search in each database were as follows: (((DM or “diabetes mellitus” or diabet*) and (neuropathy or neuropath* or neurolog* or neuralgia)) or ((PDPN or “painful diabetic peripheral neuropath*” or DPNP or “diabetic peripheral neuropathic pain” or DPN or “diabetic peripheral neuropath*” or PDN or “peripheral diabetic neuropath*” or DSPN or “distal symmetric polyneuropath*”) or (“diabetic polyneuropath*” or “diabetic sensorimotor polyneuropath*” or “distal symmetric sensorimotor polyneuropath*” or “diabetic distal sensorimotor polyneuropath*”) or (“diabetic focal neuropath*” or “diabetic multifocal neuropath*” or “diabetic amyotroph*” or “diabetic autonomic neuropath*” or “symmetric diabetic proximal motor neuropath*” or “diabetic mononeuropath*”))) and (duloxetin* or cymbalta or irenka or “drizalma sprinkle” or ariclaim or xeristar or yentreve). In addition, the reference lists of the included studies were manually searched for additional eligible studies.

### Study selection and quality assessment

After removing duplicate studies, 2 reviewers (CS Wu and YR Huang) independently reviewed the titles and abstracts of the eligible studies. Non-RCTs and non-eligible studies were excluded (upon reviewer consensus) from further analyses. Subsequently, the full texts of the included articles were analyzed. Both reviewers independently assessed the risk of bias by using the Cochrane risk-of-bias tool for RCTs (RoB 2.0) [31]. Any disagreement between the 2 reviewers was mitigated by a third reviewer.

### Data collection and analysis

Odds ratio (OR) and 95% confidence interval (CI) values were calculated using the Mantel–Haenszel method for dichotomous outcome data, whereas the mean difference (MD) and 95% CI values were calculated through inverse variance weighting for continuous data. Outcome data, such as sample size, mean, and standard deviation (SD), were extracted for each treatment. If the included articles reported only standard error values, the corresponding SD values were calculated using relevant software. Heterogeneity was investigated using the *I*^2^ statistic, and 25%, 50%, and 75% values indicated low, moderate, and high degrees of heterogeneity, respectively [32]. The pooled estimates of the MD and OR values were calculated using random-effects models. In addition to reporting the overall effects of duloxetine, we also have done subgroup analyses of the effects of different doses of duloxetine on primary outcomes of reduction in patients’ weekly mean pain scores. Statistical significance was set at *P* < 0.05. The meta-analysis was performed using RevMan (version 5.4) [33].

## Results

### Included articles

**Fig. 1:**
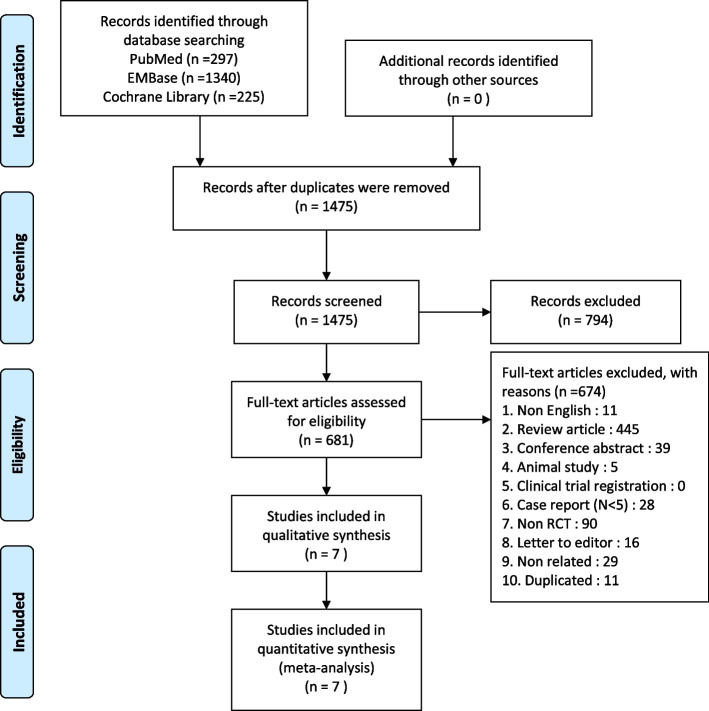
Flowchart of study selection

Our search returned a total of 1862 relevant articles; of them, 1475 were excluded after the removal of duplicates. Next, 794 studies were excluded after the titles and abstracts were screened. The full texts of the remaining 681 articles were reviewed, and 674 of them were eliminated on the basis of the exclusion criteria. Finally, a total of 7 eligible RCTs [34–40] were included in our meta-analysis. Figure 1 depicts the flowchart of study selection.

### Study characteristics

The 7 included studies comprised a total 2205 patients (men, 1246 [56.5%]; mean age, 60.2 years). Patients received 20, 40, 60, and 120 mg of duloxetine per day. Six studies had a follow-up duration of 12 weeks, and one study had a follow-up duration of 8 weeks [39]. Reduction of 24-h weekly mean pain score (daily scores were recorded in a diary) was reported as the primary outcome in 6 studies and the reduction in 24-h average BPI pain score (scores recorded during weekly visits) in one study. Dropout rates were between 13.6% and 25.7%, which were not sufficiently high to affect the statistical power. Table 1 presents a summary of the characteristics of the included studies.

**Table 1:** Characteristics of the included studies

**Characteristics**				**Participants**			**Intervention**	**Comparison**	**Outcome**				
**Source**	Study design	Country	Jadad score	Diagnosis	Age/Case no	Female No.(%)	DLX	PLC	Primary outcome	DLX	PLC	Drop-out rate, %	Severe AE DLX/PLC, No
							Case no./dose/frequency/duration	Case no./dose/frequency/duration		Baseline pain score, Mean ± SE			
**Gao et al., 2015 **[40]	Double-blind, RCT	China	3	1. Daily pain > 6 mos 2. Michigan score ≥ 3 3. BPI weekly pain ≥ 4	61.4/405	223 (55.1%)	203/60 mg/QD/12 wks	202/PLC/QD/12 wks	Weekly mean 24-h avg. pain score	5.7 ± 1.7	5.6 ± 1.7	13.80%	3/2
**Rowbotham et al., 2012 **[39]	Double-blind, rct	USA	5	1. Daily pain > 6 mos 2. Michigan score ≥ 3 3. Weekly 24-h pain score ≥ 4	59.9/108	48 (44.4%)	57/60 mg/QD/8 wks	51/PLC/QD/8 wks	Weekly mean 24-h avg. pain score	6.61 ± 1.37	6.62 ± 1.23	-	1/1
**Gao et al., 2010 **[38]	Double-blind, rct	China	4	1. Daily pain > 6 mos 2. Michigan score ≥ 3 3. Weekly 24-h pain score ≥ 4	59.3,215	114 (53.0%)	109/60 mg or 120 mg/QD/12 wks	106/PLC/QD/12wks	BPI avg. pain	5.5 ± 1.3	5.5 ± 1.4	13.60%	2/2
**Yasuda et al., 2011 **[34]	Double-blind, rct	Japan	4	1. Daily pain > 6 mos 2. DSPN diagnostic criteria in Japan	60.8/338	82 (24.3%)	40 mg: 85/40 mg/QD/12 wks	167/PLC/QD/12 wks	Weekly mean 24-h avg. pain score	40 mg: 5.79 ± 1.23	5.78 ± 1.17	16.70%	5/6
							60 mg: 86/60 mg/QD/12 wks			60 mg: 5.76 ± 1.17			
**Wernicke et al., 2006 **[35, 41]	Double-blind, RCT	USA	5	1. Daily pain > 6 mos 2. Michigan score ≥ 3 3. Weekly 24-h pain score ≥ 4	60.7/334	130 (38.9%)	60 mg: 114/60 mg/QD/12 wks	108/PLC/QD/12 wks	Weekly mean 24-h avg. pain score	60 mg: 6.1 ± 1.6	5.9 ± 1.4	25.70%	7/5
							120 mg: 112/60 mg/BID/12 wks			120 mg: 6.2 ± 1.5			
**Raskin et al., 2005 **[36]	Double-blind, RCT	Canada	5	1. Daily pain > 6 mos 2. Michigan score ≥ 3 3. Weekly 24-h pain score ≥ 4	58.8/348	186 (53.4%)	60 mg: 116/60 mg/QD/12 wks	116/PLC/QD/12 wks	Weekly mean 24-h avg. pain score	60 mg: 5.5 ± 1.1	5.5 ± 1.3	15%	6/4
							120 mg: 116/60 mg/BID/12 wks			120 mg: 5.7 ± 1.3			
**Goldstein et al., 2005 **[37]	Double-blind, RCT	USA	5	1. Daily pain > 6 mos 2. Michigan score ≥ 3 3. Weekly 24-h pain score ≥ 4	60.1/457	176 (38.5%)	20 mg: 115/20 mg/QD/12 wks	115/PLC/QD/12 wks	Weekly mean 24-h avg. pain score	20 mg: 5.9 ± 1.6	5.8 ± 1.5	24.70%	Total 19
							60 mg: 114/60 mg/BID/12 wks			60 mg: 6.0 ± 1.7			
							120 mg: 113/60 mg/BID/12 wks			120 mg: 5.9 ± 1.4			

*Abbreviations*: *RCT* randomized control trial, *DLX* duloxetine, *PLC* placebo, *No* number, *BPI* Brief Pain Inventory, *Wks* weeks, *Mos* months, *Avg* average, *QD* quaque die, *SE* standard error, *AE* adverse event, *DSPN* distal symmetric polyneuropathy

### Outcomes

#### Pain improvement

**Fig. 2:**
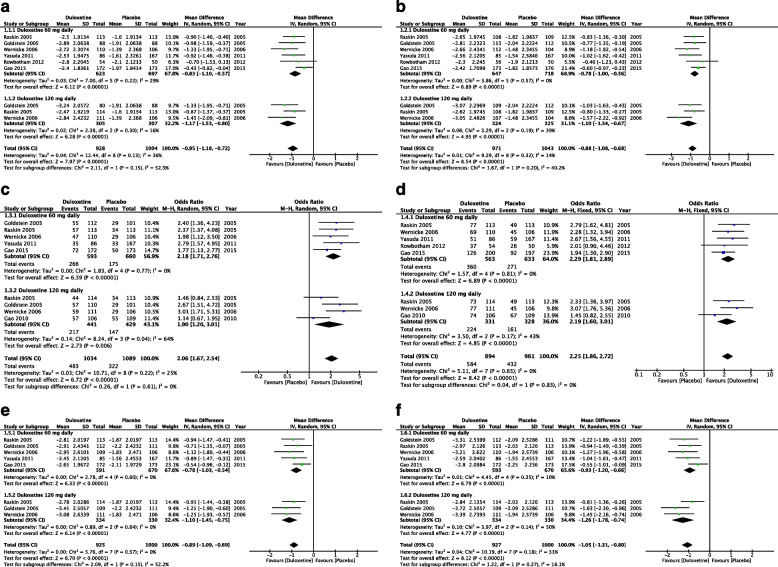
Pain. **A** Mean improvement in the weekly average of patients’ 24-h pain scores on an 11-point Likert-type scale at ≤ 12 weeks. **B** Mean improvement in pain severity assessed using the Brief Pain Inventory scale: average pain scores at ≤ 12 weeks. **C** Number of patients with ≥ 50% improvement in the weekly average of 24-h pain scores on the 11-point Likert-type scale at ≤ 12 weeks. **D** Number of patients with ≥ 30% improvement in the weekly average of 24-h pain scores on the 11-point Likert-type scale at ≤ 12 weeks. **E** Mean improvement in patients’ night pain scores on the 11-point Likert-type scale at ≤ 12 weeks. **F** Mean improvement in patients’ worst pain scores on the 11-point Likert-type scale at ≤ 12 weeks

A total of six studies reported improvements in weekly mean pain scores. Duloxetine was more efficacious than placebo, with low to moderate heterogeneity noted across studies (MD =  − 0.95, 95% CI =  − 1.18 to − 0.72, *Z* = 7.79, *P* < 0.00001, and *I*^2^ = 36%; Fig. 2A). Subgroup analysis of different doses of duloxetine indicated significant improvements with doses of 40, 60, and 120 mg but not with that of 20 mg (MD =  − 0.45, 95% CI =  − 1.05 to 0.15, and *P* = 0.14). Head-to-head comparisons between the effective doses revealed no significant differences.

Six studies reported improvements in BPI average pain scores, and overall, duloxetine was significantly more efficacious than placebo, with low heterogeneity observed across studies (MD =  − 0.88, 95% CI =  − 1.08 to − 0.68, *Z* = 8.54, *P* < 0.00001, and *I*^2^ = 14%; Fig. 2B).

In total, 6 studies reported the numbers of patients with a 50% pain reduction. Overall, the efficacy of duloxetine was higher than that of a placebo, with low heterogeneity noted across studies (OR = 2.06, 95% CI = 1.67 to 2.54, *Z* = 6.72, *P* < 0.00001, and *I*^2^ = 25%; Fig. 2C).

A total of 6 studies reported a number of patients with a 30% pain reduction. Duloxetine was significantly more efficacious than placebo, with low heterogeneity observed across studies (OR = 2.25, 95% CI = 1.86 to 2.72, *Z* = 8.42, *P* < 0.00001, and *I*^2^ = 0%; Fig. 2D).

Improvements in night pain scores were reported in a total of 5 studies. The efficacy of duloxetine was higher than that of a placebo, with low heterogeneity noted across studies (MD =  − 0.89, 95% CI =  − 1.09 to − 0.69, *Z* = 8.70, *P* < 0.00001, and *I*^2^ = 0%; Fig. 2E).

In total, 5 studies reported improvements in worst pain scores. Duloxetine was significantly more efficacious than placebo, with low to moderate heterogeneity noted across studies (MD =  − 1.05, 95% CI =  − 1.31 to − 0.80, *Z* = 8.12, *P* < 0.00001, and *I*^2^ = 31%; Fig. 2F).

#### Patient-reported health performance and quality of life

**Fig. 3:**
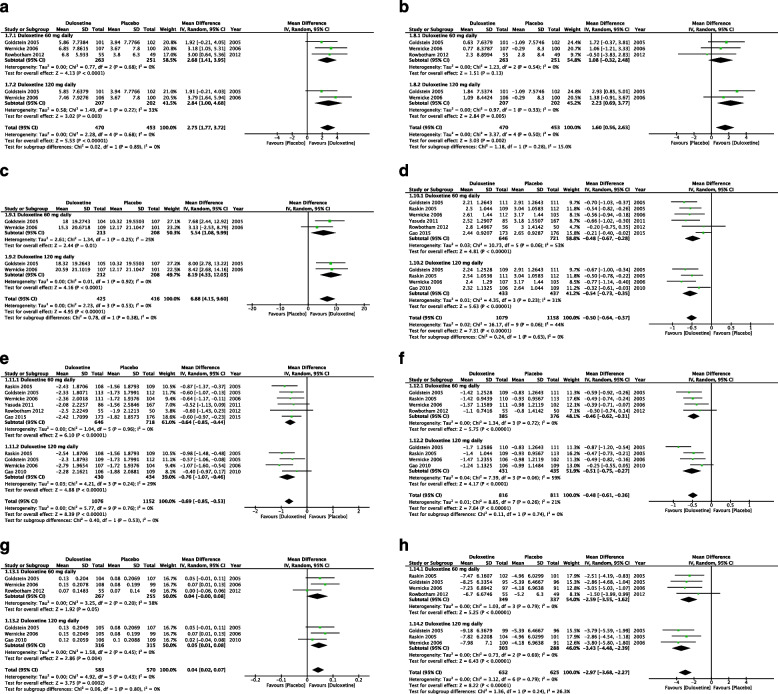
Quality of life. **A** Mean improvement in patients’ scores on the Short Form-36 (SF-36) physical functioning domain at ≤ 12 weeks. **B** Mean improvement in patients’ scores on the SF-36 mental health domain at ≤ 12 weeks. **C** Mean improvement in patients’ scores on SF-36 bodily pain domain at ≤ 12 weeks. **D** Patients’ scores on the Patient Global Impression of Improvement scale at ≤ 12 weeks. **E** Mean improvement in patients’ scores on the Brief Pain Inventory (interference) scale: average of the scores on 7 items at ≤ 12 weeks. **F** Mean improvement in patients’ scores on the Clinical Global Impression severity subscale at ≤ 12 weeks. **G** Mean improvement in patients’ scores on the European Quality of Life Instrument 5D version at ≤ 12 weeks. **H** Mean improvement in patients’ scores on the Short-Form McGill Pain Questionnaire sensory component at ≤ 12 weeks

Improvements in the physical functioning, mental health, and bodily pain domains of the SF-36 questionnaire were reported by a total of 3, 3, and 2 studies, respectively. For all domains, duloxetine exhibited higher levels of efficacy than did placebo, with low heterogeneity across studies for all domains (physical functioning: MD = 2.75, 95% CI = 1.77 to 3.72, *Z* = 5.53, *P* < 0.00001, and *I*^2^ = 0% (Fig. 3A); mental health: MD = 1.60, 95% CI = 0.56 to 2.63, *Z* = 3.03, *P* = 0.002, and *I*^2^ = 0% (Fig. 3B); and bodily pain: MD = 6.88, 95% CI = 4.15 to 9.60, *Z* = 4.95, *P* < 0.00001, and *I*^2^ = 0% (Fig. 3C)).

Improvements in patients’ scores on the PGI-I, BPI (interference subscale), CGI (severity subscale), EQ-5D, and SF-MPQ (sensory component) tools were reported by 7, 7, 5, 4, and 4 studies, respectively. Overall, the efficacy of duloxetine in improving the aforementioned scores was significantly higher than that of the placebo, with low to moderate heterogeneity observed among studies (PGI-I: MD =  − 0.50, 95% CI =  − 0.64 to − 0.37, *Z* = 7.31, *P* < 0.00001, and *I*^2^ = 44% (Fig. 3D); BPI: MD =  − 0.69, 95% CI =  − 0.85 to − 0.53, *Z* = 8.39, *P* < 0.00001, and *I*^2^ = 0% (Fig. 3E); CGI: MD =  − 0.48, 95% CI =  − 0.61 to − 0.36, *Z* = 7.64, *P* < 0.00001, and *I*^2^ = 21% (Fig. 3F); EQ-4D: MD = 0.04, 95% CI = 0.02 to 0.07, *Z* = 3.75, *P* = 0.0002, and *I*^2^ = 0% (Fig. 3G); and SF-MPQ: MD =  − 2.97, 95% CI =  − 3.68 to − 2.27, *Z* = 8.22, *P* < 0.00001, and *I*^2^ = 0% (Fig. 3H)).

### Safety and compliance

**Fig. 4:**
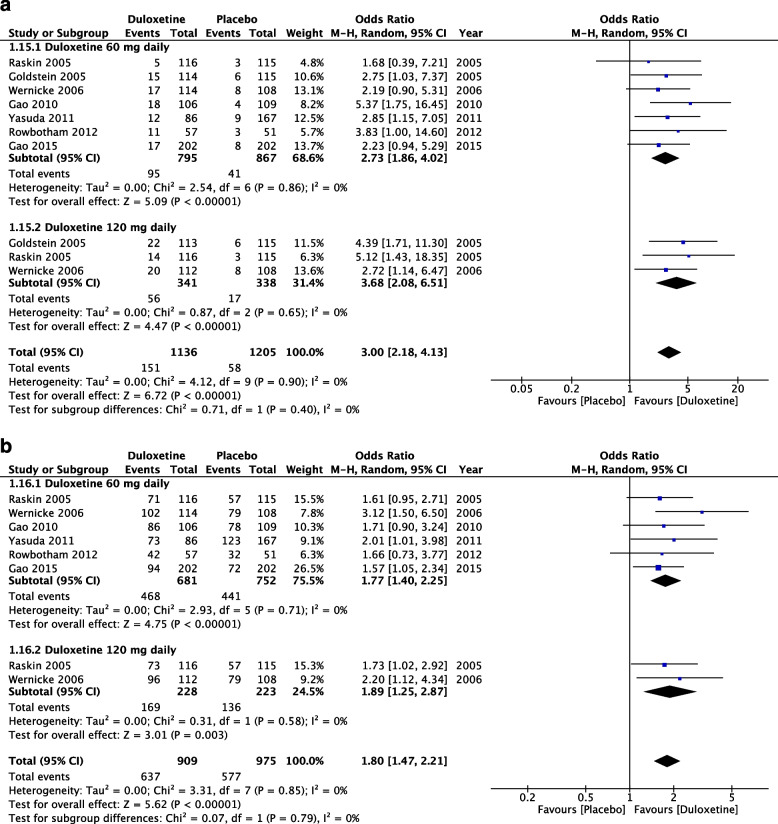
Adverse events. **A** Numbers of participants who dropped out from the studies because of adverse events. **B** Numbers of participants with at least a single adverse event

A total of 7 studies reported a number of patients who dropped out because of adverse events. The risk associated with duloxetine was significantly higher than that associated with placebo, with low heterogeneity noted across studies (OR = 3.00, 95% CI = 2.18 to 4.13, *Z* = 6.72, *P* < 0.00001, and *I*^2^ = 0%; Fig. 4A).

In total, 6 studies reported a number of patients with at least 1 adverse event. Duloxetine was associated with significantly higher levels of risk than was placebo, with low heterogeneity observed across studies (OR = 1.80, 95% CI = 1.47 to 2.21, *Z* = 5.62, *P* < 0.00001, and *I*^2^ = 0%; Fig. 4B).

A total of 5 studies reported the numbers of all adverse events. We analyzed the 6 most common adverse events associated with duloxetine (Table 2). Nausea had the highest incidence rate (20.21%; 10.4 to 30.2%), followed by somnolence (12.73%; 8.4 to 21.6%), dizziness (10.10%; 5.8 to 15.1%), fatigue/malaise (8.14%; 5.0 to 12.4%), constipation (8.01%; 5.0 to 12.8%), and decreased appetite (2.89%; 5.4 to 10.4%). The numbers of serious adverse events, defined as events leading to prolonged hospitalization, life-threatening experience, severe disability, or death during the study, reported in the 7 included studies were also recorded (Table 1). No significant differences were observed between duloxetine and placebo; moreover, the studies reported no common severe adverse event, except hyperglycemia, which was reported by a total of 3 studies [36, 37, 40] and electrolyte imbalance, which was reported by a total of 2 studies [35, 39].

**Table 2:** Adverse events reported by the included studies

	**Gao (2015) **[40], **(*n* = 202), *n***	**Gao (2010) **[38]**, (*n* = 106), *n***	**Yasuda (2011) **[34]**, (*n* = 171), *n***	**Wernicke (2006) **[35, 41]**, (*n* = 226), *n***	**Rowbotham (2012) **[39]**, (*n* = 57), *n***	**Total (*n* = 762), *n* (%)**
**Nausea**	21	32	24	68	9	154 (20.21%)
**Somnolence**	17	17	37	26	-	97 (12.73%)
**Dizziness**	17	16	10	30	4	77 (10.10%)
**Fatigue/malaise**	10	8	9	28	7	62 (8.14%)
**Constipation**	10	11	11	29	-	61 (8.01%)
**Decreased appetite**	11	11	-	-	-	22 (2.89%)

### Study quality

**Fig. 5:**
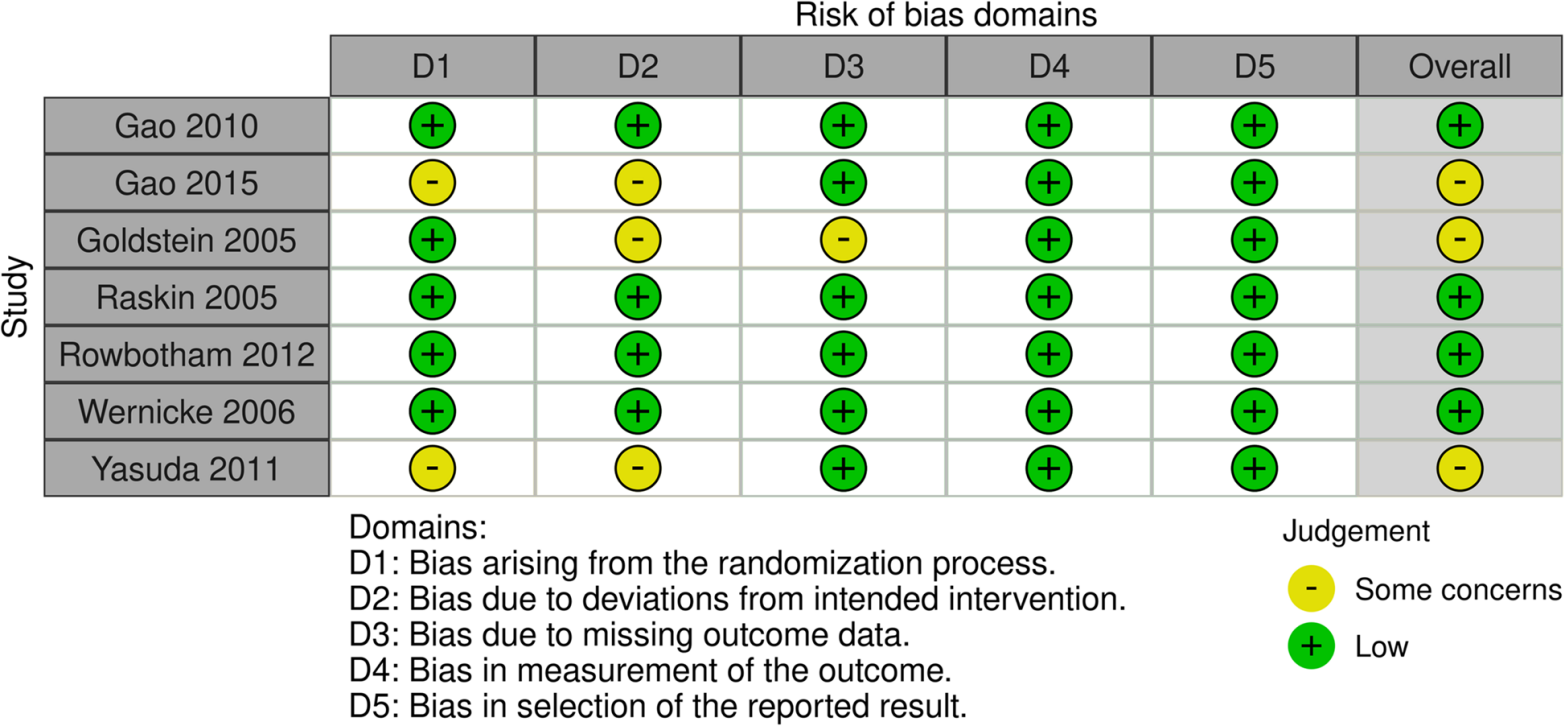
Risk of bias of the included studies

The quality of the included studies was assessed using RoB 2.0; the details are presented in Fig. 5 (ROB) and the supplementary information file. RoB 2.0 is an outcome-based instrument, but since each study’s outcome measures do not have different potential risks of bias (they all have the same assessment process and are all subjective scales), the evaluation is presented within a single study-based figure. A total of 4 studies were classified as low risk and 3 studies (conducted by Goldstein et al. [37], Yasuda et al. [34], and Gao et al. [40]) indicated some concerns. The study by Goldstein et al. had some concerns in RoB 2.0 domains 2 and 3, which was based on a high dropout rate of 24.7% and significant differences between the treatment (duloxetine: 60 mg/day, 13.2%; 120 mg/day, 19.5%) and placebo (5.2%) groups (*P* < 0.001). The study by Yasuda 2011 had some concerns in domains 1 and 2 because allocation concealment was not explained clearly. The study conducted by Gao et al. incompletely described the processes of randomization sequence generation and allocation concealment, leading to some concerns in domains 1 and 2.

## Discussion

The efficacy of duloxetine in treating PDPN has been demonstrated in a total of 4 RCTs conducted between 2005 and 2011 [34–37]. In total, 3 sequential 52-week-long phase studies have revealed that duloxetine is superior to routine care in the long-term management of PDPN [41–43]. By contrast, another 52-week-long study reported no significant difference between PDPN and routine care in terms of their efficacy in pain control; nevertheless, the findings confirmed the long-term safety of duloxetine [44]. Other medications for PDPN include antiepileptic agents, pregabalin, and gabapentin and certain antidepressants (including tricyclic antidepressants) [45]. However, most of these medications are only partially effective for PDPN; moreover, they are frequently discontinued because of their associated adverse events, which limit their clinical utility.

As mentioned, duloxetine and pregabalin are the only 2 FDA-approved drugs for PDPN. Some studies have reported that the efficacy of duloxetine in PDPN pain relief is non-inferior [46, 47] to or even better than that of pregabalin [48, 49]. Only 3 recent systemic reviews and meta-analyses included RCTs on duloxetine for PDPN. Of them, 2 studies included a total of 5 studies [50, 51] and 1 study included a total of 6 studies [22]. Unfortunately, no relevant meta-analyses have been conducted since 2017. In addition to assessing pain severity and PGI scores, which were analyzed in an earlier meta-analysis [43], in the present study, we assessed patients’ health performance and quality of life in terms of night pain, worst pain, and SF-36 (physical functioning, mental health, and bodily pain domains), CGI, BPI (7-item interference), EQ-5D, and SF-MPQ (sensory component) scores. To the best of our knowledge, the present study is the first meta-analysis to explore the incidence rates of common adverse events.

Compared with placebo, duloxetine significantly improved patients’ pain scores on every item; the total weighted mean (TWM) of the reduction in the weekly mean of 24-h pain scores was − 2.62 (MD − 0.89; *P* < 0.00001). The TWM values of the reductions in the weekly mean values of average pain scores on BPI, night pain scores, and 24-h worst pain scores were –2.64 (MD − 0.84 and *P* < 0.00001), − 2.80 (MD − 0.82 and *P* < 0.00001), and − 3.02 (MD − 1.00 and *P* < 0.00001), respectively. Furthermore, 45.6% and 64.5% of all patients experienced 50% and 30% pain reduction, respectively, which highlights the clinical efficacy of duloxetine in the treatment of PDPN. Duloxetine considerably improved the physical and mental health and the quality of life of patients with PDPN, as evident from their scores on the following tools: CGI (TWM =  − 1.39), PGI-I (TWM = 2.47), EQ-5D (TWM = 0.13), SF-36 (physical functioning and mental health domains, respectively: TWM = 6.06 and 1.01), and SF-MPQ (TWM =  − 7.80). (All these analyses included the 20 mg and 40 mg subgroups data).

A total of 4 doses of duloxetine were compared in the present study. The 20-mg dose improved only the weekly worst pain and CGI scores of patients with PDPN; this dose exhibited the lowest efficacy in the aforementioned 2 items. This finding implies that the clinical value of the 20-mg dose is low. By contrast, the 40-mg dose markedly improved the patients’ scores on all pain relief items and PGI-I; in terms of efficacy, this dose was non-inferior to the 60- and 120-mg doses. The 40-mg dose achieved 50% pain reduction in most patients. Although only a single study included the 40-mg dose, the aforementioned finding suggests that the 40-mg dose of duloxetine can serve as an alternative to the 60- and 120-mg doses. The 60- and 120-mg doses exhibited significant efficacy in almost all items (except for the low efficacy of the 60-mg dose in improving patients’ SF-36 mental health and EQ-5D scores), and no significant differences were observed between them. However, the 120-mg dose was associated with numerically better outcomes than the 60-mg dose in terms of pain reduction (weekly mean of 24-h pain, BPI average pain, night pain, and worst pain), and health performance, and quality of life. Thus, the 120-mg dose of duloxetine may be useful when the 60-mg dose fails to ensure adequate relief in patients with PDPN.

Duloxetine was found to be safe in the treatment of PDPN: the incidence of severe adverse events was low, not higher than that associated with placebo treatments. However, complications such as nausea, somnolence, dizziness, fatigue, constipation, and decreased appetite were common; because of these common adverse events, approximately 12.6% of patients dropped out of the studies. Approximately, 71.3% of all patients experienced at least 1 adverse event. Therefore, when administering duloxetine, patients must be informed about these adverse effects, and they must be closely monitored for the incidence and severity of common adverse events. Symptomatic interventions should be administered as required.

The present study has some limitations. We found only 1 additional eligible study [40] compared with the last relevant meta-analysis [22]. Nevertheless, because the sample size of the additional RCT was high (second largest among the 7 included studies; *n* = 405) and comprehensive data on pain relief were reported by the RCT, we could provide considerable additional and updated data regarding the efficacy and safety of duloxetine in the treatment of PDPN. Of the studies included in the present study, the study by Gao et al. (2010) [29] reported variable dosage schedules for duloxetine depending on clinical responses (60 or 120 mg); this made it challenging for us to perform subgroup analyses. Hence, we performed the analyses for benefit considering that all patients received the higher dose (120 mg) and for harm considering that if all patients received the lower dose (60 mg). We further performed sensitivity analysis by excluding the data of the aforementioned study from each item, and the results remained significant. All 7 studies had similar cohorts and good uniformity in terms of the use of assessment tools. Although recent studies have demonstrated a high efficacy of duloxetine, further studies are warranted to evaluate the effects of various duloxetine doses, particularly the 40-mg dose. Furthermore, head-to-head RCTs on pregabalin, gabapentin, and SNRI drugs are necessary to identify the optimal treatment option for patients with PDPN.

## Conclusions

The findings of our systematic review and meta-analysis suggest that duloxetine is more efficacious than placebo treatments in terms of pain relief and improvements in the quality of life of patients with PDPN. Duloxetine is safe, as indicated by the rarity of severe adverse events; however, patients receiving this drug should be monitored for some common complications, such as nausea, somnolence, dizziness, fatigue, constipation, and decreased appetite. This study may serve as a reference for future studies aimed at developing suitable interventions and optimal treatment strategies for patients with PDPN.

## Supplementary Information

**Additional file 1.**

## Data Availability

The data sets produced and analyzed during the present study are available from the corresponding author on reasonable request.

## Abbreviations

PDPN: Painful diabetic peripheral neuropathy
SNRI: Serotonin–norepinephrine reuptake inhibitor
BPI: Brief Pain Inventory
SF-36: Short form-36
PGI-I: Patient’s Global Impression of Improvement
CGI: Clinical Global Impression scale
EQ-5D: European Quality of Life Instrument 5D version
SF-MPQ: Short-Form McGill Pain Questionnaire
RCT: Randomized controlled trial
MD: Mean difference
OR: Odds ratio
SD: Standard deviation
SE: Standard error
TWM: Total weighted mean
AE: Adverse event
