# Supplementary material for: Efficacy and safety of duloxetine in painful diabetic peripheral neuropathy: a systematic review and meta-analysis of randomized controlled trials
Source: Syst Rev. 2023 Mar 21;12:53. doi: 10.1186/s13643-023-02185-6 (PMC10031998; doi:10.1186/s13643-023-02185-6)
Supplement: Supplementary file 1 — Additional file 1. [file 13643_2023_2185_MOESM1_ESM.docx]

| Bias | Authors’ judgement | Support for judgement |
| --- | --- | --- |
| Goldstein et al. 2005 | | |
| Domain 1: Risk of bias arising from the randomization process | Low risk | “Patients (457) were randomly assigned to … as determined by a computer-generated random sequence using an interactive voice response system (IVRS).” “The IVRS was used to assign blister cards containing study drug to each patient confirmed through IVRS entry of a confirmation number found on the card.” |
| Domain 2: Risk of bias due to deviations from the intended interventions | Some concerns | Double-blindness was kept throughout the study  Although the analysis was by ITT, the dropout rate was 24.7% and significant difference were found between treatment groups and placebo groups, with the duloxetine 60mg/d (13.2%), 120 mg/d (19.5%), and the placebo group (5.2%; P<0.001). The discontinuations were probably related to adverse events of duloxetine. |
| Domain 3: Missing outcome data | Some concerns | Although the analysis was by ITT, the dropout rate was 24.7% and significant difference were found between treatment groups and placebo groups, with the duloxetine 60mg/d (13.2%), 120 mg/d (19.5%), and the placebo group (5.2%; P<0.001). The discontinuations were probably related to adverse events of duloxetine. |
| Domain 4: Risk of bias in measurement of the outcome | Low risk | Double-blind  Patients in both groups were seen weekly for the first 5 weeks of treatment and then bi-weekly thereafter. |
| Domain 5: Risk of bias in selection of the reported result | Low risk | All items of outcome were reported. |
| Other bias | Low risk | Sponsored by Eli Lilly and Company. |
| Raskin et al. 2005 | | |
| Domain 1: Risk of bias arising from the randomization process | Low risk | “Assignment to treatment groups was determined by a computer-generated random sequence using an Interactive Voice Response System”  “Patients received … capsules of duloxetine hydrochloride or placebo capsules identical to duloxetine capsules. ” |
| Domain 2: Risk of bias due to deviations from the intended interventions | Low risk | Double-blindness was kept throughout the study |
| Domain 3: Missing outcome data | Low risk | Dropout rate 15%, analysis was by ITT. |
| Domain 4: Risk of bias in measurement of the outcome | Low risk | Double-blind The diary, vital signs, and adverse events were collected in both groups at each subsequent visit |
| Domain 5: Risk of bias in selection of the reported result | Low risk | All outcomes were reported. |
| Other bias | Low risk | Sponsored by Eli Lilly and Company. |
| Wernicke et al. 2006 | | |
| Domain 1: Risk of bias arising from the randomization process | Low risk | “Assignment to a treatment group was determined by a computer-generated random sequence using an interactive voice response system (IVRS).” “The IVRS was used to assign blister cards containing study drug to each patient. ” |
| Domain 2: Risk of bias due to deviations from the intended interventions | Low risk | Double-blindness was kept throughout the study  Dropout rate 25.7%, but no significant difference was found among groups (dropout rate: 60 mg Dul. - 25%), 120mg Dul. - 30% , placebo - 21%). Analysis was by ITT. |
| Domain 3: Missing outcome data | Low risk | Dropout rate 25.7%, but no significant difference was found among groups (dropout rate: 60 mg Dul. - 25%), 120mg Dul. - 30% , placebo - 21%). Analysis was by ITT. |
| Domain 4: Risk of bias in measurement of the outcome | Low risk | Double-blind  The outcome assessment was measured by corresponding scales completed by the patients in diaries in both groups. |
| Domain 5: Risk of bias in selection of the reported result | Low risk | Reported outcomes were in accordance with the pre-specified analysis plan. |
| Other bias | Low risk | Sponsored by Eli Lilly and Company. |
| Gao et al. 2010 | | |
| Domain 1: Risk of bias arising from the randomization process | Low risk | “12 weeks of double-blind, placebo-controlled treatment ” “Patients who met entry criteria were enrolled … randomly assigned 1:1 to either flexible dose duloxetine or placebo.”  “Study medication was provided in capsules containing … duloxetine hydrochloride as enteric-coated pellets or matching placebo.” |
| Domain 2: Risk of bias due to deviations from the intended interventions | Low risk | Double-blindness was kept throughout the study  Dropout rate 13.6%. Analysis was by ITT. “Missing data were imputed using last-observation-carried-forward (LOCF) approach.” |
| Domain 3: Missing outcome data | Low risk | Dropout rate 13.6%. Analysis was by ITT. “Missing data were imputed using last-observation-carried-forward (LOCF) approach.” |
| Domain 4: Risk of bias in measurement of the outcome | Low risk | Double-blindness was kept throughout the study |
| Domain 5: Risk of bias in selection of the reported result | Low risk | All measures reported. |
| Other bias | Low risk | Sponsored by Eli Lilly and Company. |
| Yasuda et al. 2011 | | |
| Domain 1: Risk of bias arising from the randomization process | Some concerns | “Before randomization, an assigning table was prepared using Create Key Code 3.3. Patients were randomly assigned to duloxetine 40 or 60 mg or placebo groups in a 1:1:2 ratio…” “multicenter, randomized, double-blind, placebo-controlled, group-comparison phase III study”  The concealment was not clearly explained. |
| Domain 2: Risk of bias due to deviations from the intended interventions | Some concerns | “multicenter, randomized, double-blind, placebo-controlled, group-comparison phase III study”  The concealment was not clearly explained. |
| Domain 3: Missing outcome data | Low risk | Dropout rate 16.7%. ”For patients who discontinued the study, the aforementioned assessments were collected at their last visit.” |
| Domain 4: Risk of bias in measurement of the outcome | Low risk | Both groups measured the efficacy through diary and visited as scheduled. |
| Domain 5: Risk of bias in selection of the reported result | Low risk | All measures reported. |
| Other bias | Low risk | Sponsored by Eli Lilly and Company. |
| Rowbotham et al. 2012 | | |
| Domain 1: Risk of bias arising from the randomization process | Low risk | “…patients were randomized 1:1 to each treatment arm via an interactive voice response system using a randomization schedule that was generated before study start. ” “To maintain blinding, subjects assigned to duloxetine took 2 pills daily: 1 duloxetine 60 mg and 1 placebo. ” |
| Domain 2: Risk of bias due to deviations from the intended interventions | Low risk | Double-blindness was kept throughout the study. |
| Domain 3: Missing outcome data | Low risk | Low dropout rate 3.7% |
| Domain 4: Risk of bias in measurement of the outcome | Low risk | Double-blindness was kept throughout the study. |
| Domain 5: Risk of bias in selection of the reported result | Low risk | All measures reported. |
| Other bias | Low risk | None identified |
| Gao et al. 2015 | | |
| Domain 1: Risk of bias arising from the randomization process | Some concerns | “Randomised (1:1) to one of two treatment groups, patients received either duloxetine 60 mg once daily or placebo once daily.” “phase 3, multicenter, randomised, double-blind, parallel, placebo-controlled trial”  The concealment was not clearly explained. |
| Domain 2: Risk of bias due to deviations from the intended interventions | Some concerns | “phase 3, multicenter, randomised, double-blind, parallel, placebo-controlled trial”  The concealment was not clearly explained. |
| Domain 3: Missing outcome data | Low risk | Dropout rate 13.8% (60 mg Dul. - 14.8%, placebo - 12.9%).  “… discontinuation were not significantly different between treatment groups.”  Analysis was by ITT. |
| Domain 4: Risk of bias in measurement of the outcome | Low risk | Both groups measured the efficacy through diary and visited as scheduled. |
| Domain 5: Risk of bias in selection of the reported result | Low risk | Outcomes listed in the clinical trial registration were all reported. |
| Other bias | Low risk | Sponsored by Eli Lilly and Company. |
